# Characterization of lymphocyte subsets over a 24-hour period in Pineal-Associated Lymphoid Tissue (PALT) in the chicken

**DOI:** 10.1186/1471-2172-7-1

**Published:** 2006-01-11

**Authors:** Jeffrey A Mosenson, John A McNulty

**Affiliations:** 1 Department of Cell Biology, Neurobiology and Anatomy, Loyola University Stritch School of Medicine, Maywood, IL 60153, USA

## Abstract

**Background:**

Homeostatic trafficking of lymphocytes in the brain has important relevance to the understanding of CNS disease processes. The pineal gland of the chicken contains large accumulations of lymphocytes that suggest an important role related to homeostatic circadian neuro-immune interactions. The purpose of this initial study was to characterize the lymphocyte subsets in the pineal gland and quantitate the distribution and frequency of lymphocyte phenotypes at two time points over the 24-hour light:dark cycle.

**Results:**

PALT comprised approximately 10% of the total pineal area. Image analysis of immunocytochemically stained sections showed that the majority of lymphocytes were CD3^+ ^(80%) with the remaining 20% comprising B-cells and monocytes (Bu-1^+^), which tended to distribute along the periphery of the PALT. T-cell subsets in PALT included CD4^+ ^(75–80%), CD8^+ ^(20–25%), TCRαβ/Vβ_1_^+ ^(60%), and TCRγδ^+ ^(15%). All of the T-cell phenotypes were commonly found within the interfollicular septa and follicles of the pineal gland. However, the ratios of CD8^+^/CD4^+ ^and TCRγδ^+^/TCRαβ/Vβ_1_^+ ^within the pineal tissue were each 1:1, in contrast to the PALT where the ratios of CD8^+^/CD4^+ ^and TCRγδ^+^/TCRαβ/Vβ_1_^+ ^each approximated 1:4. Bu-1^+ ^cells were only rarely seen in the pineal interstitial spaces, but ramified Bu-1^+ ^microglia/macrophages were common in the pineal follicles. Effects of the 24-h light:dark cycle on these lymphocyte-pineal interactions were suggested by an increase in the area of PALT, a decline in the density of TCRαβ/Vβ_1_^+ ^cells, and a decline in the area density of Bu-1^+ ^microglia at the light:dark interphase (1900 h) compared to the dark:light interphase (0700 h).

**Conclusion:**

The degree of lymphocyte infiltration in the pineal suggests novel mechanisms of neuro-immune interactions in this part of the brain. Our results further suggest that these interactions have a temporal component related to the 24-hour light:dark cycle and that CD8^+ ^and TCRγδ^+ ^T-cells are preferentially recruited to the pineal follicles. Pineal microglia/macrophages were common and represent an important candidate for mediating these lymphocyte-pineal interactions via secretion of cytokines and chemokines.

## Background

The view that the central nervous system (CNS) is an immunologically privileged site has been widely accepted based on classic experiments such as those of Medawar [1], who showed that rabbit skin allografted into the CNS failed rejection. This immunologic privilege is specifically related to the low levels of MHC class II expression [2], which is responsible for antigen presentation in the brain. An additional mechanism for maintaining immune privilege is the presence of the blood-brain barrier (BBB), which is a semi-permeable barrier, composed of endothelial cells that restrict the interface between the brain and immune circulatory products.

Specific regions of the brain, the circumventricular organs, lie outside the BBB [3] and are therefore subject to the effects of serum-derived substances including immune factors. One of those regions is the pineal gland, which has been studied extensively in recent years for its immuno-regulatory function [4-7]. The gland secretes a potent hormone, melatonin, that tends to stimulate the immune system and its production of cytokines [5-8]. Because the pineal gland's neuroendocrine functions are closely linked to the 24-hour light:dark cycle [9], these pineal-immune interactions are also thought to have a temporal component. Evidence for effects of the pineal gland on photoperiodic changes in the immune system has been collected in both birds and mammals [10-13].

Interactions between the neuroendocrine and immune systems presume that feedback mechanisms are present from one system to the other via immune soluble factors. Antigenic stimulation [14], inflammation [13] and treatment with cytokines [15,16] have all been shown to modulate the neuroendocrine functions of the gland, although the cellular and molecular mechanisms of this feedback are still poorly understood. One cell population in the pineal gland that has been implicated as mediators of these immune effects on pinealocyte functions are the microglia/macrophages. These cells regulate pinealocyte neurite length and serotonin content in an invitro system and they upregulate cytokine expression, MHC class II and other surface antigens in response to cytokines and bacterial wall components [17-19].

Feedback of the immune system on the pineal gland is further indicated by reports of accumulations of lymphocytes in the pineal of both avian and mammalian species [2,20-25]. In some species such as the chicken, these accumulations of lymphocytes account for up to 30% of the total volume of the gland [20]. The large size of this pineal-associated lymphoid tissue (PALT) and the extent of lymphocyte infiltration suggest novel mechanisms of neuro-immune interactions in this part of the brain. The presence of PALT raises several important questions regarding the mechanisms of interactions between lymphocytes and the pineal gland, including effects on circadian rhythms in both the immune system and the pineal gland. To date, there are no studies on possible changes in the composition and distribution of lymphocytes in the pineal over a light: dark cycle. Accordingly, the purpose of this study was to further characterize the phenotypes and distribution of lymphocytes in the chicken pineal gland, and to determine if lymphocyte subsets vary over the 24-h light: dark cycle.

## Results

### General histology of the pineal gland

**Figure 1:**
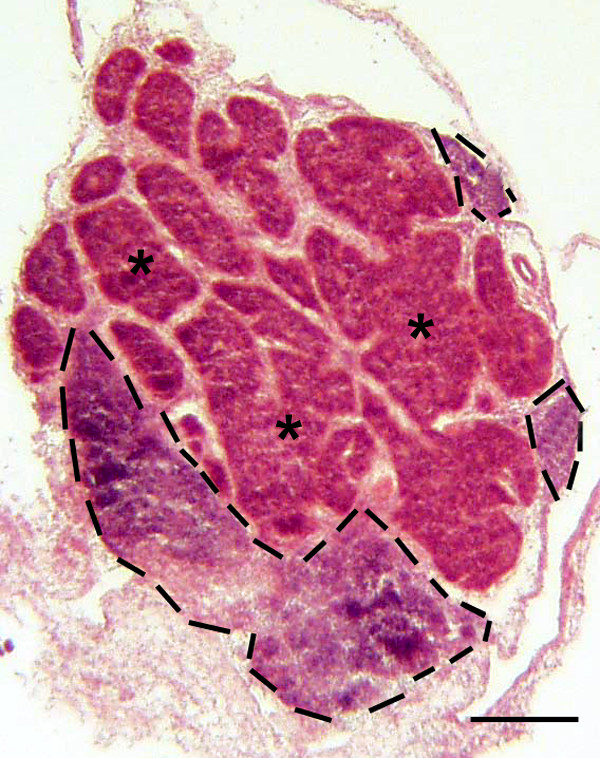
**Histology of the pineal gland**. Frozen section of the pineal gland stained with H&E. The pineal follicles (*) comprise pinealocytes and supportive cells arranged as epithelium. Prominent interstitial septa separate individual follicles. Several large accumulations of mononuclear (MN) cells, forming the PALT (bordered by the dashed black lines) are seen. Bar indicates 200 μm.

The pineal gland in the chicken develops through the formation of a series of vesicles or follicles separated by prominent septa (Fig. 1) as previously described [20-23,26]. Each follicle has a central lumen into which the apical ends of the pinealocytes protrude. The interstitial spaces contain connective tissue, smaller capillaries and mononuclear (MN) cells, which occurred either singly or in small clusters. PALT was typically located along the periphery of the gland, but could be found centrally betweem adjacent follicles. For descriptive purposes, PALT refers to the larger accumulations of mononuclear cells with distinct borders separating it from the pineal tissue.

**Figure 2:**
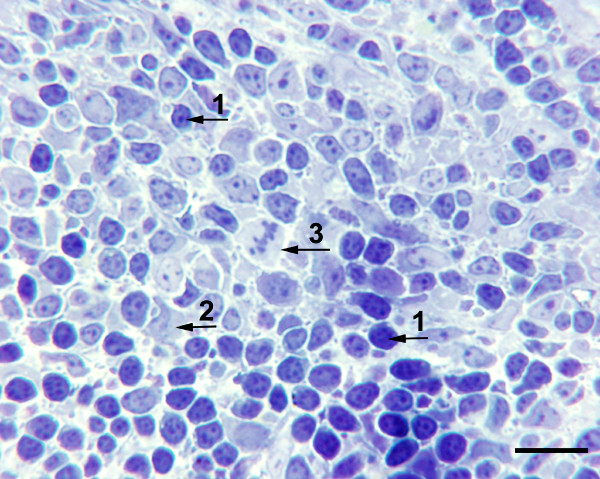
**High magnification of PALT**. Toluidine-blue stained 1 μm epoxy section of PALT at higher magnification showing numerous small lymphocytes (1), and larger pleomorphic cells (2). Mitotic figures were common (3). Bar indicates 10 μm.

Most of the cells within PALT resembled small lymphocytes, although larger, pleomorphic cells were also present (Fig. 2). These mononuclear cells were also frequently seen infiltrating the pineal follicle (see below). Mitotic figures were commonly observed throughout the PALT (Fig. 2), inter-follicular spaces and even within the pineal follicles.

### Lymphocyte phenotypes and distributions

**Figure 3:**
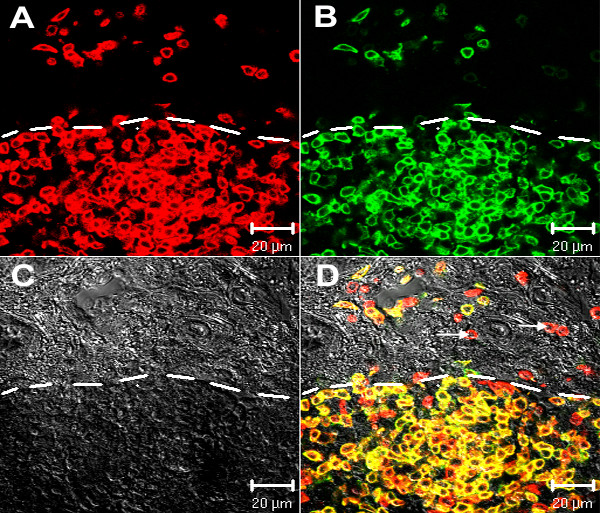
**Distribution of CD3^+ ^and CD4^+ ^cells**. PALT stained with anti-CD3 (panel A) and anti-CD4 (panel B). Note that the majority of the lymphocytes are double positive for CD3 and CD4 (yellow in Panel D). Several CD3^+^/CD4^- ^cells are seen primarily along the periphery of the PALT and bordering a pineal follicle (arrows, Panel D). The DIC image (Panel C) shows the border of the PALT (white dashed line).

Immunohistochemical phenotyping of lymphocytes within the PALT and pineal tissue showed that the majority of lymphocytes stained positively for CD3 (Fig. 3). CD3^+ ^cells were also commonly seen within the pineal inter-follicular septa bordering PALT or within the gland at some distance from the PALT.

Two-color immuno-staining revealed that the majority of CD3^+ ^lymphocytes were also CD4^+ ^(Fig. 3). Fewer CD3^+ ^lymphocytes in the PALT co-localized with CD8α. These CD3^+^CD8α^+ ^cells tended to distribute randomly throughout the PALT, although on occasion were seen localized along the periphery of the PALT. Antibodies to both TCR1 (γδ) and TCR2 (αβ/Vβ_1_) showed staining that co-localized with CD3-positive lymphocytes, although the frequency of TCRγδ^+ ^cells was significantly less than the frequency of TCRαβ/Vβ_1 _^+ ^cells. The TCRγδ^+ ^cells tended to be found along the periphery of the PALT.

**Figure 4:**
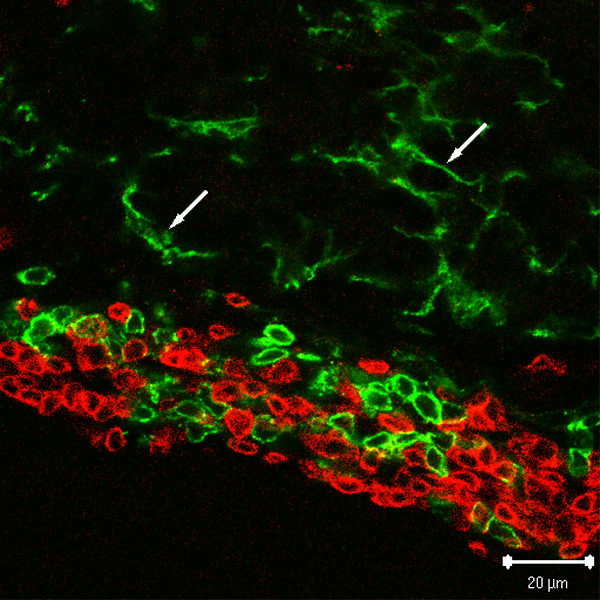
**Distribution of Bu-1^+ ^cells**. PALT is seen bordering a pineal follicle stained with anti-CD3 (red) and anit-Bu-1 (green). The arrows indicate processes of microglia, which are morphologically distinct from the round lymphocytes in PALT.

Bu-1^+ ^cells were present within PALT and did not co-localize with CD3 (Fig. 4). Overall, there were few Bu-1^+ ^cells compared to CD3^+ ^cells. Similar to the γδ-positive cells, Bu-1^+ ^cells were found typically along the periphery of the PALT. On two occasions, clusters of Bu-1^+ ^cells surrounded by CD3^+ ^cells were seen within the PALT. Staining with antibodies against IgM and Igλ (light chain) revealed positive cells, with denser staining from Igλ. However, there was also extensive labeling within the lumen of follicles and the interstitial spaces, possibly due to soluble antibodies. This background staining negated the effectiveness of the anti-IgM and anti-Igλ antibodies for use in image analysis of lymphocyte phenotypes.

Bu-1^+ ^cells resembling microglia/macrophages were also seen within the pineal follicles (Fig. 4). The microglia/macrophages were easily distinguished by their ramified morphologies. In contrast to the common occurrence of T-cell lymphocyte phenotypes in the interstitial spaces between follicles, round, mononuclear Bu-1^+ ^cells were rarely seen in these compartments.

**Figure 5:**
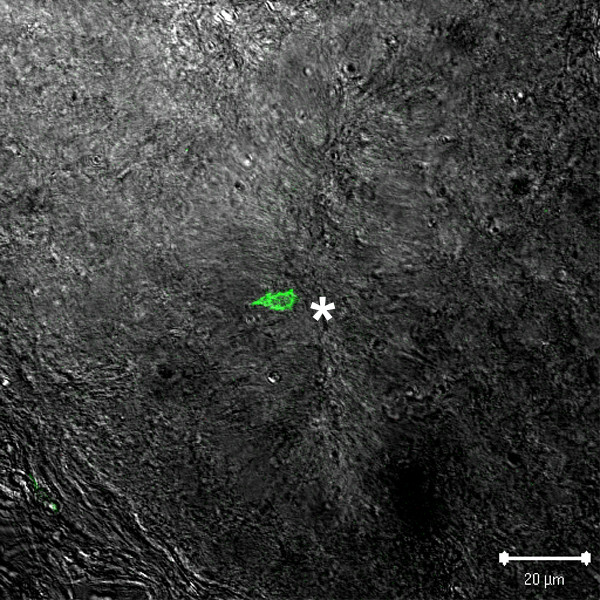
**Lymphocyte within a pineal follicle**. A TCRγδ^+ ^cell is seen bordering the central lumen (*) of a pineal follicle.

Large numbers of T-lymphocytes were found within the interfollicular spaces occurring either singly or in small clusters. The T-cells were also observed within the pineal follicles, even bordering the central lumen (Fig. 5). The frequency of CD4^+ ^cells approximated the frequency of CD4^-^/CD8^+ ^cells within the interfollicular spaces.

### Image analysis of lymphocyte phenotypes

Measurements of the profile areas of the PALT and pineal tissue showed that PALT made up approximately 7–12% of the total pineal area. The average profile area of PALT in glands collected at the light: dark interphase was approximately 104,000 μm^2 ^whereas the glands collected at the dark: light interphase was approximately 51,000 μm^2^. In contrast, the average profile area of pineal tissue did not change between the two time points studied (81 × 10^4 ^μm^2 ^vs. 77 × 10^4 ^μm^2^).

**Figure 6:**
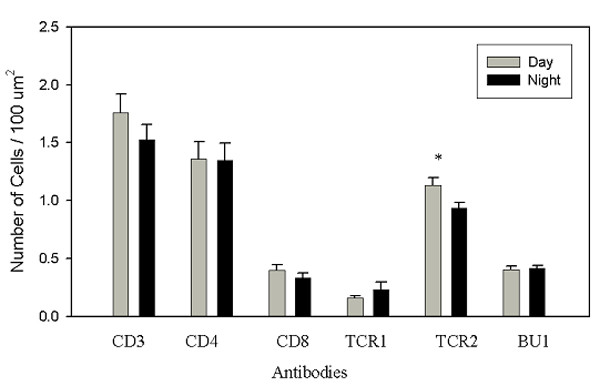
**Quantitation of lymphocyte phenotypes in PALT**. Means (+sem) of the number of positive cells/area for each phenotype at the time points sampled. Day = dark:light interphase; Night = light:dark interphase. Differences between means (p < 0.05) are indicated by asterisk. Day N = 10, Night N = 10.

Image analysis of lymphocyte phenotypes in PALT revealed that approximately 80% of positively stained mononuclear cells were CD3^+ ^T cells with the remaining 20–25% comprising BU-1^+ ^cells (B cells, monocytes). Estimates of the number of cells per unit area confirmed that the majority of CD3^+ ^cells were CD4^+ ^(approximately 75–80%) and αβ^+ ^(approximately 60%) when all glands were combined. CD8α^+ ^cells accounted for approximately 20% of the CD3^+ ^cells. The TCRγδ^+ ^cells had the lowest frequency (approximately 15% of the CD3^+ ^cells). The only phenotype that showed a significant difference between the two time points studied were the TCRαβ/Vβ_1 _^+ ^cells, which exhibited an 18% decline (p < 0.05) at the light: dark interphase (Fig. 6).

**Figure 7:**
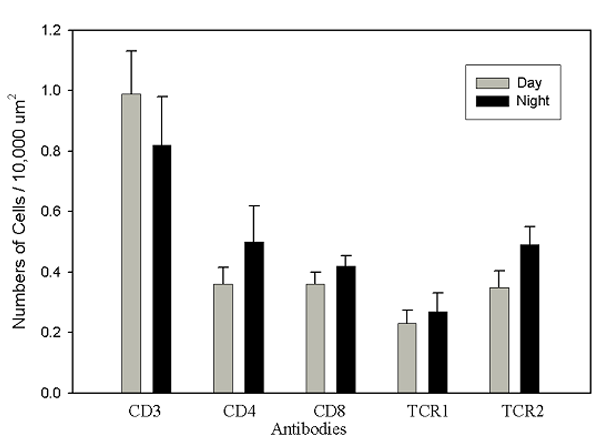
**Quantitation of lymphocyte phenotypes in non-PALT compartments**. Means (+sem) of the number of positive cells/area for each phenotype at the time points sampled. Bu-1^+ ^lymphocytes were not detectable in this compartment. Note differences in the scale compared to Figure 6.

Each of the phenotypes of T-lymphocytes was observed in the interstitial spaces as well as within the pineal follicles. As noted above, Bu-1^+ ^cells were only rarely seen in the interstitial spaces, but Bu-1^+ ^microglia/macrophages were common in the pineal follicles. In contrast to the PALT, where the ratio of CD4^+^/CD8α^+ ^cells was about 4:1 (Fig. 6), the ratio of CD4^+^/CD8α^+ ^cells in the non-PALT compartments was approximately 1:1 (Fig. 7). Similarly, there was approximately a 4-fold difference in the ratio of TCRαβ/Vβ_1_^+^/TCRγδ^+ ^cells for PALT compared to the non-PALT regions of the gland (Figs. 6, 7). There were no significant differences in the numerical density of lymphocyte phenotypes between the two time points studied in the non-PALT regions, but it is noteworthy that the density of TCRαβ/Vβ_1_^+ ^cells increased 40% at the light: dark interphase (p < 0.10), which was opposite to the trend for this phenotype in the PALT.

BU-1-positive cells were common in the pineal tissue, although B cells could not be readily distinguished from the microglia/macrophages, which are also stained with this antibody. Immuno-staining with anti-IgM and anti-Igλ (light chain) did not resolve the B cells because these antibodies stained secretory products throughout the pineal tissue, including the lumen of follicles. Because Igλ labels most of the immunoglobulin fraction (e.g., light chain), it was observed to stain more intensely than IgM.

The day: night differences in the area density (μm^2^/μm^2^) of the Bu-1^+ ^microglia/macrophages within pineal tissue approached significance as analyzed by a two-tailed Student's *t*-test (p < 0.07, n = 10 for each time point) with a 43% decline at the light: dark interphase (0.020 ± SEM 0.003) compared to the dark: light interphase (0.035 ± SEM 0.007).

## Discussion

The histology of the PALT in this study was in general agreement with previous reports [20-23]. Moreover, the present finding that PALT occupied approximately 10% of the total pineal area in 14–21 day old chicks was reasonably close to Cogburn's [20] estimate that PALT occupied 18% of the total volume in older White Leghorn chickens. The phenotypes and distributions of lymphocytes in PALT resembled the gut-associated lymphoid tissues (GALT) found in mucosal structures such as the proventriculus [27]. PALT and GALT each showed a higher concentration of T cells centrally (mostly CD4^+ ^and TCRαβ/Vβ_1_^+^), whereas TCRγδ^+ ^and Bu-1^+ ^cells were found more peripherally.

Our results are consistent with the hypothesis that lymphocyte-pineal interactions vary over the light:dark cycle. The 2-fold change in profile area of PALT and the decline in density of TCRαβ/Vβ_1 _^+ ^cells at the light:dark interphase suggest that lymphocyte trafficking in the PALT varies with time. However, additional samples are required over the 24-h light:dark cycle as well as other criteria (e.g., free-running under constant darkness) need to be met before a circadian rhythm in pineal lymphocyte trafficking can be definitively established in this system. Because immunohistochemistry allows analysis of only a small percentage of the tissue in any gland, we are undertaking to develop methodologies of fluorescence activated cell sorting (FACS) to analyze lymphocyte subsets in the gland.

Cogburn and Glick [20] concluded that PALT formation is dependent on the bursa and thymus based on the reduction in size of PALT following bursectomy and/or thymectomy with whole body irradiation. In a later study, Cogburn and Glick [21] showed that tritium-labelled lymphocytes from the thymus and bursa migrated to the pineal gland, but only in older (4–5 week old) chicks. Studies are currently underway to determine whether soluble chemotactic factors from chicken pineal glands induce migration of specific lymphocyte subtypes using an invitro migration assay. This specificity of migration is suggested by the present study showing that the ratios of CD8^+ ^to CD4^+^, and TCRγδ^+ ^to TCRαβ/Vβ_1 _^+ ^cells are nearly four fold greater in the pineal tissue compared to that found in PALT. The close correlation of CD8 to TCRγδ is supported by Chen et al. [28] who reported that the majority of TCRγδ cells expressed CD8. A preferential recruitment of CD8^+ ^and TCRγδ^+ ^cells into the pineal tissue was also found to occur in the retina [29], which is notable considering that the pineal gland shares homologies related to common photoreceptive functions [30]. Imagawa et al. [29] speculated that the lymphocytes played a role in the normal surveillance of the retina and functioned to remove cell debris as well as remaining in the retina as memory T cells.

The scarcity of Bu-1^+ ^cells in the inter-follicular (non-PALT) compartment is further evidence of the preferential recruitment of lymphocytes to the pineal tissue. Image analysis of these cells revealed that they comprised 20–25% of PALT lymphocytes, similar to that found in the spleen [31,32]. Cogburn and Glick [21] described a much higher proportion of B lymphocytes (42%) based on a cytotoxic assay in older (6 weeks) chicks of a different strain (New Hampshire). However, Olah and Magyar [26], using the Bu-1b antibody in the New Hapmpshire strain showed staining patterns that were similar to those observed in the present study. As reported here, the Bu-1-positive cells were predominantly found along the periphery of PALT and the authors also described large clusters of Bu-1-positive cells, which they described as germinal centers [26].

Lymphocyte interactions with pinealocytes are clearly implicated by their common occurrence within the pineal follicles, which in some cases exhibited heavy infiltration [22], even as far as the central lumen of the pineal follicles (see Fig. 5). The proliferation of these cells in the non-PALT pineal compartments is greater than in the PALT based on tritiated-thymidine incorporation studies [22,26] and counts of mitotic figures (unpublished observations). The enhanced proliferation of lymphocytes in the pineal tissue (non-PALT) suggests that the pineal cells produce blastogenic products that may also affect the differentiation of the lymphocytes [22]. These results are additional evidence that lymphocytes traffic regularly between PALT and the pineal follicles.

The Bu-1^+ ^cells observed within the pineal follicles are most likely microglia/macrophages because their distribution and ramified morphology resemble the microglia/macrophages described in the mammalian pineal gland [2,3,17,33-36]. To our knowledge, this is the first description of these cells in the avian pineal gland and, as such, represent ideal candidates as cellular interfaces between the immune system and the neuroendocrine pinealocytes. In contrast to most other parts of the brain, microglia/macrophages in the pineal gland are constitutively active as evidenced by the expression of MHC class II [2,3,17,33-37]. Because these cells are actively involved in antigen presentation, and secrete potent cytokines (e.g., IL1), they have been implicated in the regulation of pinealocyte differentiation and neuroendocrine functions. Tsai and McNulty [36] demonstrated in the rat that pineal microglia regulate pinealocyte neurite length and the production of serotonin in vitro. The production of melatonin could also be stimulated in vivo by treatment with IL-1β [15], presumably produced by these activated microglia/macrophages. More recently, Jiang-Shieh et al. [17] demonstrated that pineal microglia/macrophages are in constant surveillance of blood-borne pathogens and presumably other serum-derived substances.

Day-night differences in the expression of the Bu-1 epitope in microglia/macrophages are consistent with their hypothesized role as interfaces in the interaction with PALT lymphocytes. Increased expression of Bu-1 occurred at the dark:light interphase (0700 h), the time immediately following when cells were exposed to the highest levels of melatonin, which is produced during the dark phase (cf [38]). The time-point corresponded to the rise in area density of TCRαβ/Vβ_1 _^+ ^cells in the PALT. This temporal relationship may be explained by increased secretion of microglial cytokines and chemokines assuming that increased expression of Bu-1 represents activation of the microglia.

The terminology used to describe microglia in the pineal gland is complicated by the fact that this organ is outside the blood-brain barrier. Although Perry and Gordon [39] described similar populations of cells in the choroid plexus and meninges as macrophages, we include the term "microglia" because the pineal gland is part of the brain, a terminology adapted by other authors [17,33,34,40]. However, it is not clear whether these cells are undifferentiated microglia or resident macrophages derived from circulating monocytes. In addition to B-cells, the Bu-1 antibody stains cells of the monocyte line and recognizes a highly glycosylated transmembrane protein that has no similarity to known mammalian markers [31,41,42]. The Bu-1 antibody has also been shown to stain microglia within the sensory epithelium of the ear [42].

The functional significance of lymphocyte trafficking in the pineal gland has yet to be determined, although there are two overall working hypotheses, which are not mutually exclusive. One hypothesis addresses the mechanisms involved in the recruitment of lymphocytes to the gland and their possible differentiation to form distinct subpopulation(s), which are involved in immunological surveillance of the CNS including the cerebrospinal fluid (CSF). In many species, the gland and the PALT are juxtaposed to the choroid plexus, which produces CSF. Lymphocytes have been shown to migrate through the choroid plexus and enter the CSF at these locations [20]. The second hypothesis addresses immunological mechanisms regulating the neuroendocrine functions of the gland. As noted above, several studies have shown that immune-derived cytokines modulate both neural and neuroendocrine activities. It is therefore conceivable that lymphocyte trafficking in the pineal regulates the circadian production of melatonin. This potential circuit could represent a direct feedback of the immune system on a gland that is known to regulate immune functions.

## Conclusion

In conclusion, this study has provided a detailed quantitative description of pineal-associated lymphocyte phenotypes at two time points over the 24-hour light:dark cycle. Our results show that lymphocytes comprise a significant component of pineal tissue in this species. The data further suggest that CD8^+^/ TCRγδ^+ ^cells are preferentially recruited to the pinealocytes and that pineal-lymphocyte interactions are dynamically linked to the light:dark cycle. Pineal microglia/macrophages are proposed to play an important role in these interactions through their secretion of cytokines and chemokines. The unique accumulation of lymphocytes in this part of the brain has important functional implications with regard to homeostatic mechanisms of neuro-immune interactions, especially those involving circadian components of both the immune and nervous systems and the possible role of these cells in immune surveillance in other parts of the CNS. Further studies of the cellular and molecular mechanisms of these homeostataic pineal-lymphocyte interactions will provide a better understanding of disease processes in the nervous system.

## Methods

### Animals

Male White Leghorn chickens (3 days old) were purchased commercially (Ideal Poultry, Texas) between June and September, and raised to 2–3 weeks of age under standard laboratory conditions in a fully accredited animal care facility according to recommendations in the guide for the care and use of laboratory animals, and the guidelines of the Institutional Animal Care and Use Committee at Loyola University Stritch School of Medicine. The photoperiod in the animal room was controlled by automatic timers programmed for a 12:12 light: dark cycle. The intensity of the light at the level of the cages was approximately 450 lux. Chicks were killed by rapid decapitation following CO_2 _inhalation. Glands were collected at the dark: light interphase (0700 h) and 12 h later at the light: dark interphase (1900 h) just after lights on and prior to lights off, respectively. These time points were selected because the 0700 h time point represents the delayed effects of greatest exposure to the pineal hormone melatonin, which is produced during the dark phase. The 1900 h time point represents the delayed effects of least exposure to melatonin, which is not produced during the daytime. A total of 10 animals were collected at each time point.

### Preparation of tissue sections

The pineal glands were fixed in cold 4% paraformaldehyde for 1 h along with thymus, bursa of Fabricius, and spleen, which were used as control tissues. The tissues were washed in PBS (2×, 10 min) and the pineal glands cleaned of any attached meninges or vessels before being placed in the cryoprotectant (30% sucrose solution) for 2 h. Tissues were then placed in *TBS Tissue Freezing Medium *using a penny to determine the orientation of the pineal, frozen on dry ice and stored at -80°C. Tissue blocks were serially cut at a thickness of 6 μm on the cryomicrotome with the pineal glands sectioned from caudal to rostral. The sections were transferred to Superfrost Plus slides and stored at -80°C until staining.

### Histology

Every eighth pineal section (48 μm intervals) was stained with haematoxylin and eosin (H&E). These sections were used to determine the location of the PALT, and to measure the profile areas of the pineal gland and the PALT that corresponded to the nearest sections used for immunocytochemistry. Semi-thin (1 μm), toluidine blue stained epoxy sections from a previous study [43] were also used to evaluate the histology of the PALT.

### Immunohistochemistry

Immunohistochemical methods followed previously published procedures [18,44]. Optimal conditions for immunohistochemical staining with each monoclonal antibody (mAb) were determined using spleen, thymus and bursa. Prior to staining, pineal sections were selected based on the presence of PALT as determined from H&E sections. These sections were brought to room temperature (RT) and briefly washed with PBS. A solution of Superblock, Triton-X, and 3% goat serum was added for 30 min at RT. Following PBS wash (2X), the sections were stained for 30 min with mAbs in a solution of BSA/ PBS (2 g/1000 ml) at RT. Mouse hybridoma supernatants were supplied by Dr. Chen-lo Chen (University of Alabama, Birmingham). The following clones (1:5 dilution) were used: CT-3 (anti-CD3), CT-4 (anti-CD4), CT-8 (anti-CD8α), TCR-1 (anti-TCRγδ), TCR-2 (anti-TCRαβ/Vβ_1_), M-1 (anti-IgM) and L1 (anti-Igλ light chain). The TCR-3 (anti-TCRαβ/Vβ_2_) was not used for analysis in this study because it gave a diffuse staining pattern that was inconsistent with previously published findings. Commercially purified mAb's (SouthernBiotechnology, Birmingham, AL) (1:100 dilution) for CD4 and CD8α were used to verify specificity of the supernatants. A separate anti-CD3 mAb (rat, 1:100 dilution, Abcam) was used to delineate the PALT and to determine co-localization with other T-cell markers. The Bu-1 antibody (AV20 clone, SouthernBiotechnology) was used to detect B cells and cells in the monocyte lineage. Following primary antibody incubation, sections were washed 3× in PBS for 10 min at 4°C. The secondary antibody, FITC labelled goat anti-mouse at a dilution of 1:250, or a mixture of FITC labelled goat anti-mouse at a dilution of 1:250 and Alexia-555 labelled goat anti-rat (Molecular Probes) at a dilution of 1:100 was added for 30 min at RT in the dark. Sections reacted with mouse IgG1 isotype controls were included with each staining. Other controls included serial dilutions of the primary antibody and omission of the primary antibody. Immunohistochemical images were captured with a laser scanning confocal microscope (Zeiss LSM 510).

### Image analysis

Because the optical slice thickness (1.3 μm) of the confocal microscope was less than the thickness of the section (6 μm), all images were collected from the middle of the section as determined by focusing through the section. All confocal microscope parameters (e.g., optical slice, detector gain, detector offset) were kept constant during collection of all images. Every PALT was digitized using a 63X lens making every effort to minimize the amount of overlap from image to image.

After converting the files to 8-bit grayscale images in Photoshop (Adobe) they were imported to Scion Image (Scion Corp) software. This software was used to measure area profiles of pineal tissue and PALT from the H&E sections and area profiles of PALT from the diffraction interference contrast (DIC) images generated by the Zeiss confocal instrument. The area of immunoreaction product was measured after thresholding to determine the optical density that corresponded to specific immunostaining for each antibody as described previously [35]. Area densities of immunostaining were calculated by dividing the area of immunoreaction product by the total area of reference (e.g., PALT). To convert area density of immunoreaction product to number of cells per area, a factor was calculated that represented the average area of reaction product per cell. Briefly, for each antibody, the average area of reaction product was calculated from 20 separate fields and divided by the average number of PALT lymphocytes in those same fields. Dividing the immunoreaction area densities by cell factors for each antibody provided estimates of the number of positive cells per unit area.

The number of positive cells found outside the PALT (i.e., within the interlobular pineal septa and follicles) were counted directly from sections by two observers. For these counts, cells along the periphery of the gland and nearest to PALT were not included.

Area density of Bu-1^+ ^staining on ramified cells within the pineal follicles was determined from 3 random fields for each gland. As described above for lymphocytes, the threshold in Scion Image was set to the optical density that corresponded to specific immunostaining of these Bu-1^+ ^cells.

### Statistics

Data are expressed as mean ± SEM. Mean values were calculated for each antibody for each animal and these means pooled according to time of sacrifice (n = 10/time point). Differences between grouped means were determined by a two-tailed Student's t-test. A difference of p < 0.05 was considered significant.

## Authors' contributions

JAMo collected and analyzed the data, and wrote the paper. JAMc originated the idea for the research, oversaw the collection and analysis of data, and revised the manuscript.
